# Influence of Glycomacropeptide on Rehydration Characteristics of Micellar Casein Concentrate Powder

**DOI:** 10.3390/foods10081960

**Published:** 2021-08-23

**Authors:** Ram R. Panthi, Francesca Bot, James A. O’Mahony

**Affiliations:** School of Food and Nutritional Sciences, University College Cork, T12TP07 Cork, Ireland; ram.panthi@ucc.ie (R.R.P.); francesca.bot@ucc.ie (F.B.)

**Keywords:** glycomacropeptide, micellar casein concentrate, powder rehydration

## Abstract

Glycomacropeptide (GMP) shows potential for enhancing the rehydration properties of high-protein dairy powders due to its hydrophilic nature. This study involved formulating micellar casein concentrate (MCC) solutions (8.6% final protein content) with 0, 10, and 20% GMP as a percentage of total protein, and investigated the physicochemical and rehydration properties of the resultant freeze-dried powders (P-MCC-0G, P-MCC-10G, and P-MCC-20G, respectively). The surface charges of caseins in the control MCC and 10 or 20% GMP blended solutions were −25.8, −29.6, and −31.5 mV, respectively. Tablets prepared from P-MCC-10G or P-MCC-20G powders displayed enhanced wettability with contact angle values of 80.6° and 79.5°, respectively, compared with 85.5° for P-MCC-0G. Moreover, blending of GMP with MCC resulted in faster disintegration of powder particles during rehydration (i.e., dispersibility) compared to P-MCC-0G. Faster and more extensive release of caseins from powder particles into solution was evident with the increasing proportion of GMP, with the majority of GMP released within the first 15 min of rehydration. The results of this study will contribute to further development of formulation science for achieving enhanced solubility characteristics of high-protein dairy powder ingredients, such as MCC.

## 1. Introduction

High-protein, casein-dominant powders (e.g., micellar casein concentrate, milk protein isolate, milk protein concentrate) are newer-generation ingredients with significant market potential because of their techno-functional properties such as emulsification and gelation, as well as nutritional attributes [1,2]. However, such powders generally display poor rehydration properties when dissolved in aqueous media, which limits their application as demanded by end-users and consumers [3]. Developing an understanding of advanced mechanisms during the rehydration of casein-dominant powders has been the focus of many recent studies [2,3]. Mechanistically, primary powder particles disintegrate into smaller particles, subsequently releasing caseins into solution [4]. However, prior to a complete disappearance of primary powder particles, wetting, sinking, swelling, and dispersion occur, and casein-dominant powders typically show longer dispersion times compared with conventional milk powders, such as skim milk powder [3]. Powder particles with poor rehydration characteristics interact slowly with water [5,6], which is likely influenced by the closely fused casein micelles at the powder surface [7], leading to the development of hydrophobic surface properties [8].

Recent research has shown the importance of the physicochemical properties of feed material in enhancing rehydration properties of resulting high-protein dairy powders [2]. On concentration of milk systems, the distance between casein micelles reduces considerably, and caseins are more likely to interact and fuse with nearby casein micelles [9]. The fusion of such casein material results in poor dispersibility of subsequent powders [7,10]. Therefore, formulation strategies that restrict micellar interactions show promise in enhancing rehydration properties of casein-dominant powders [11,12]. For example, micellar casein solution mixed with up to 12% sodium caseinate [12] or sodium chloride, whey proteins, citrate, or phosphate [6] showed improvement in rehydration characteristics of subsequent powders. Milk protein concentrate (MPC) dispersion mixed with nano-sized spacers containing lipid (lecithin, up to 10% of total solids) before spray drying showed enhanced solubility characteristics of resultant powder when the powders were stored for an extended period [11]. Sun et al. [13] added 0.3% salts (sodium citrate, sodium pyrophosphate, and sodium phosphate) to ultrafiltration retentate, reporting improved rehydration properties of resultant MPC powder. However, use of such additives generally impairs the processing characteristics (e.g., high viscosity), functionality (e.g., reduced casein micelle integrity) [14,15], or nutritional significance of protein enrichment. 

Glycomacropeptide (GMP), a fraction of κ-casein with 64 amino acids, is a bioactive peptide rich in branched-chain amino acids (e.g., isoleucine and valine) and free of aromatic amino acids (tryptophan, tyrosine, and phenylalanine), often used for the management of rare health conditions when phenylalanine metabolism is hindered (e.g., phenylketonuria) [16] and shows benefits for functional food applications [17]. GMP is generally glycosylated with sialic acid (N-acetyl neuraminic acid, NANA), rendering the peptide highly hydrophilic [18]. The peptide has one positively charged end, a middle segment (AA, 1–5, 17–22, 35–39, 58–65) containing mainly hydrophobic residues, and the other end bearing a negative charge due to glycosylation with sialic acid [19]. Some examples of the implications of this unique structure are that it tends to form a complex with gelatin through hydrophobic interactions [20] and protects against extensive aggregation between unfolded β-lactoglobulin at pH 6.7 [18]. Likewise, the formation of protein aggregates in solutions of sodium caseinate or denatured β-lactoglobulin was reported to be suppressed by the presence of GMP [18,21,22], providing evidence of electrostatic repulsion induced by GMP for preventing protein–protein interactions. GMP shows the tendency to self-associate at neutral pH in pure water in the presence of calcium (>1.2 mmol/g GMP) through hydrophobic interaction [23], as the negative charge of sialic acid is screened by ionic calcium (Ca^2+^) at neutral pH [24]. Consequently, it is hypothesized that GMP would enhance dissolution properties of high-protein dairy powders due to its highly hydrophilic nature; more specifically, incorporation of GMP in MCC solution at neutral pH would modulate electrostatic interactions between casein micelles, with consequent improvement of rehydration properties of subsequent high protein, casein-dominant powders. This study aimed to investigate the physicochemical properties of mixed protein systems containing MCC and GMP, as well as the rehydration characteristics of powders prepared therefrom. The results of this study will contribute to improving our understanding of the rehydration properties of high-protein dairy powders.

## 2. Materials and Methods

### 2.1. Materials

Spray-dried MCC (MCC-SD) powder was acquired from Milei (MILEI GmbH, Leutkirch im Allgäu, Germany), and spray-dried GMP powder (GMP-SD, BiPRO^®^ GMP 9000) was acquired from Agropur (Agropur Ingredients, Suite 250A, Eden Prairie, MN, USA). MCC-SD powder had a protein content of 88% (*w*/*w*, dry basis) with casein ≥92%, with reversed-phase liquid chromatography analysis showing the presence of only casein fractions, while the protein content of the GMP-SD powder sample was 85% (*w*/*w*, dry basis), with no other casein factions detected using chromatographic analysis. 

### 2.2. Preparation of Micellar Casein Concentrate and Glycomacropeptide Solutions and Powders 

MCC solutions (8.6% protein) were prepared by dissolving MCC-SD powder in ultrapure water (900 g) at 45 °C using a high shear mixture (L4-RT Laboratory Mixer, Silverson, East Longmeadow, MA, USA) at 5100 rpm for at least 30 min followed by stirring for 3 h using a magnetic stirrer at room temperature and overnight stirring at 4 °C. GMP solutions were prepared by solubilizing GMP-SD powder (12 g) in 100 g ultrapure water to yield 8.6% protein, and the solutions were magnetically stirred for 4 h at room temperature, followed by overnight stirring at 4 °C. This wet blending allowed for the replacement of 10 and 20% of total protein with GMP while maintaining total protein content constant. Experiments were designed at 0, 10, and 20% GMP addition to achieve minimal changes in casein micelle size while altering the zeta potential on the basis of data obtained from preliminary trials. After blending MCC and GMP, the pH of all solutions was adjusted to 6.9 using 1 N NaOH/HCl, after which the samples were stored overnight at 4 °C with magnetic stirring. Further pH adjustment to the samples was performed on the following day, if necessary. The prepared solutions for 0, 10, and 20% GMP protein content were identified as S-MCC-0G, S-MCC-10G, and S-MCC-20G, respectively.

The powder samples were prepared from the MCC solutions with 0, 10, and 20% of GMP protein content (P-MCC-0G, P-MCC-10G, and P-MCC-20G) using the freeze-drying technique. The obtained freeze-dried solids were converted into uniform powders using an ultracentrifugal mill operated at 6000 rpm with an 80 µm sieve (ZM 200, Retsch Centrifugal Mill, Carl Stuart Ltd., Dublin, Ireland) and stored in air-tight plastic bags at 4 °C until analyzed. 

### 2.3. Compositional Analysis of Protein Solutions and Powders

The total nitrogen content of solutions and the respective powders was determined using Kjeldahl method [25]. The nitrogen to protein conversion factors used for MCC and GMP were 6.38 and 7.07, respectively [23]. The total solids content of solutions and powder samples were determined using oven drying (103.5 °C for 5 h) [26]. Powders were dry-ashed in a muffle furnace at 800 °C for ash content determination. 

### 2.4. Particle Size and Charge in Protein Solutions

Particle size of, and charge on, casein micelles in all prepared solutions were determined using a Zetasizer Nano series HT instrument (Malvern Instruments Ltd., Worcestershire, UK). For size measurement, refractive indices for protein and dispersant (i.e., water) were set at 1.46 and 1.33, respectively. The dynamic light-scattering system utilized emission of He–Ne laser at 633 nm at the back-scattering configuration with a scattering angle of 173°. Samples were measured in triplicate at 25 °C after 120 s of temperature equilibration. 

### 2.5. Morphology of Powders

For morphological characterization, powder samples were attached to sticky rubber on an aluminum stub, which was then coated with gold and palladium (Au/Pd) up to 5 nm thickness. Images of the powder samples were taken at magnifications of 70× and 420× using a scanning electron microscope (Jeol JSM-5510, Jeol Ltd., Tokyo, Japan) operated at 5 kV.

### 2.6. Rehydration Properties of Powders

#### 2.6.1. Contact Angle of Powder Samples

Wettability of powder samples was measured using an optical tensiometer by determining the contact angle (θ) formed by a water droplet on the surface of powder tablets (Attension Theta, Biolin Scientific Ltd., Espoo, Finland). Briefly, the tablets were formed with a load of 5000 kg in a hydraulic press (Perkin Elmer, Buckinghamshire, UK) and the contact angle measurement was performed at 20 °C over 52 s recording the images of a water droplet (10 µL) spreading on the surface of the powder. The values of contact angle in the right-hand and left-hand sides were averaged during each measurement. Sample analyses were performed in triplicate on each freeze-dried powder, which was then averaged to represent the data from one sample. The contact angle data analysis for MCC powders was performed in triplicate independent trials (n = 3), while for the MCC-SD and GMP-SD samples, a total of four different samples were prepared and analyzed (n = 4) as analytical replicates. Contact angle data are expressed as an average of seven data points.

#### 2.6.2. Dispersion Characteristics of Powders

The dispersion characteristics of the powder samples were assessed optically using a Malvern Mastersizer 3000 (Malvern Instruments Ltd., Malvern, UK). Powder samples (2.5 g) were stirred in ultrapure water (200 mL) at 25 °C and 500 rpm for 240 min. Aliquots of samples were transferred into the dispensing unit of the instrument at 1, 15, 30, 45, 60, 90, 180, and 240 min. The instrument conditions selected were stirrer speed 1290 rpm, laser obscuration of 13 ± 2% with ultrapure water as dispersant. Analysis of PSD was performed using the spherical model, with particle refractive index of 1.46, absorption of 0.001, and dispersant refractive index of 1.33 [27,28]. The area under the curves for volume-based-PSD data for each primary powder peak (>1 μm) and casein micelles peak (< 1 μm) were integrated with the trapezoidal rule approach using Microsoft Excel and plotted against rehydration time.

### 2.7. Protein Release in Solution during Powder Rehydration 

Release of caseins (α-, β-, κ-casein) and GMP from primary powder particles as a function of rehydration time was assessed by measuring the protein content in supernatant obtained after centrifugation. Powder (650 mg) was dissolved in ultrapure water (50 mL) at 25 °C using a magnetic stirrer at 300 rpm. Aliquots (2 mL) of the dispersions were removed at 15, 45, and 90 min and 24 h and centrifuged at 700× *g* at 20 °C for 10 min, after which the supernatant was separated from the sediment and frozen. After thawing, the supernatant was mixed 1:1 with sample buffer and filtered using 0.20 µm nylon filters before injection on a C_18_ column (3.6 µm × 250 mm × 4.6 mm, Aeris Widepore, Phenomenex, Cheshire, UK) for reversed-phase high-performance liquid chromatography (RP-HPLC; Agilent 1220 Infinity II LC, Santa Clara, CA, USA). The composition of sample buffer and the gradient for mobile phase solvents required for generating chromatograms were as described previously [29]. Peak areas of individual milk proteins were integrated, and the data were converted to relative percentage on the basis of the area of samples measured after 24 h of rehydration. Powder rehydration was considered to be complete at 24 h, on the basis that P-MCC-0G and P-MCC-20G displayed similar particle size values after 240 min of rehydration.

### 2.8. Data Analysis

Normality and homogeneity test of variance was checked, and data were analyzed using one-way ANOVA with a Tukey test for multiple comparisons between treatments at 95% confidence level using SPSS (IBM SPSS Statistics for Windows, version 24, IBM Corp, Arnomk, NY, USA).

## 3. Results and Discussion

### 3.1. Physicochemical Properties of Protein Solutions

The physicochemical properties of MCC and GMP blended solutions are shown in Table 1. The prepared MCC solutions (S-MCC-0G, S-MCC-10G, and S-MCC-20G) had similar levels of total solids, protein, and protein on a dry basis, with values in the ranges of 9.77–9.92%, 8.64–8.58%, and 88.5–86.6%, respectively. The surface charge (zeta potential) of casein micelles in S-MCC-0G was −25.82 mV. With the inclusion of GMP in MCC at 10 or 20% of total protein in MCC solution, the negative surface charges increased to −29.6 and −32.6 mV, respectively. The more negative surface charge of the MCC-GMP blends was attributed to acidic amino acid residues and glycosylation of GMP [21], rendering the peptide highly hydrophilic; indeed, the zeta potential of 1% GMP solution at pH 6.5 has been reported to be −24.12 mV [20]. The distribution of casein micelle size in the S-MCC-0G, S-MCC-10G, and S-MCC-20G was similar (Figure S1), suggesting that casein micelle size and integrity was not influenced by higher levels of GMP in the MCC solutions.

### 3.2. Composition and Microstructure of Protein Powders

**Figure 1:**
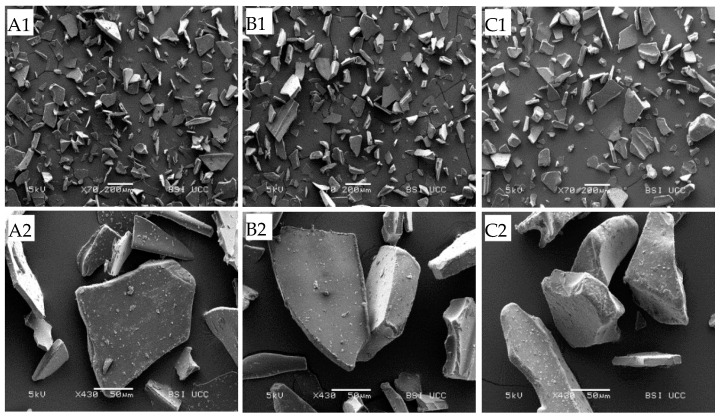
Scanning electron microscopy images (**A1**–**C1**, 70×, and **A2**–**C2**, 430×) of freeze-dried MCC powder: (**A**) (P-MCC-0G), (**B**) (P-MCC-10G), and (**C**) (P-MCC-20G) prepared from MCC solutions containing 0, 10, and 20% GMP as a percentage of total protein, respectively.

The moisture and protein contents of P-MCC-0G, P-MCC-10G, and P-MCC-20G were similar (*p* > 0.05), with moisture values of 4.0, 3.5, and 4.4% (*w*/*w*) and protein contents (*w*/*w*, dry basis) of 88.5, 85.8, and 83.8%, respectively. The ash contents (*w*/*w*, dry basis) of P-MCC-0G, P-MCC-10G, P-MCC-20G were 8.04 ± 0.04, 7.85 ± 0.09, and 7.54 ± 0.02%, respectively. The decrease in ash content with increasing proportion of GMP was partly due to lower ash content in GMP-SD (5.75%, *w*/*w*, dry basis) compared to MCC. The morphology of all powders appeared highly angular, with broken pieces evident at 70× magnification, and attachment of fine particles on the surface in those powders was revealed at magnification 430× (Figure 1). As expected, powder morphological characteristics were similar between samples regardless of GMP content, and this was attributed to the use of ultracentrifugal milling prior to analysis [29]. However, it is important to note that the morphological characteristics of freeze-dried powders differ greatly from powders produced using spray-drying, in terms of interstitial and occluded air contents and surface properties [8]. Freeze-drying was intentionally chosen in this study, as the advantage of structural uniformity in freeze-dried powders enables more accurate and direct study of the linkages between composition and rehydration properties of powders by minimizing the influence of differences in structural characteristics of powders.

### 3.3. Wettability of Micellar Casein Concentrate Powders

**Figure 2:**
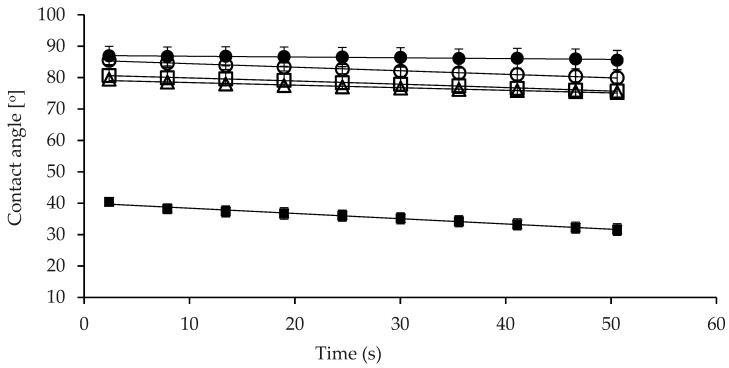
Contact angle values of water droplets on the surface of powder tablets from: ● MCC-SD, spray-dried original powder; ○ P-MCC-0G, freeze-dried MCC powder without GMP addition; □ P-MCC-10G, freeze-dried MCC prepared with 10% GMP as a percentage of total protein; Δ P-MCC-20G, freeze-dried MCC prepared with 20% GMP as a percentage of total protein; ■ GMP-SD, original spray-dried GMP powder, as a function of time. The error bars represent standard error.

Wettability of the powder samples, expressed as contact angle (θ), P-MCC-0G, P-MCC-10G, and P-MCC-20G, including the original MCC-SD and GMP-SD powders, is given in Figure 2. The contact angle data of MCC-SD and GMP-SD provide the basis for comparison between all freeze-dried MCC powders produced in this study. As expected, the MCC-SD powder had the highest θ value (86.9°) initially, decreasing slightly to 85.6°, demonstrating that the original spray-dried powder had poor wettability in water. The observed θ values were close to those for other high-protein dairy powders, such as MPC, which displayed θ values of ≈75° [28]. On the other hand, GMP-SD powder exhibited the lowest θ values (40.5°), ultimately reaching 31.5°. As GMP is highly hydrophilic, tablets prepared from GMP-SD powder interacted rapidly with water and allowed the spreading of water droplets (Figure S2). Sample P-MCC-0G, which is the freeze-dried powder of MCC solution without GMP, had a θ value 85.5° initially, which is close to that displayed by the original MCC-SD, and decreased to 80°, indicating that freeze-dried powder interacted more quickly with water compared to spray-dried original MCC-SD powder. On inclusion of 10 or 20% GMP of total protein in the samples P-MCC-10G or P-MCC-20G, the initial θ values of 80.6° and 79.5° decreased to 75.7° and 75.2°, respectively, being lower than that observed for P-MCC-0G. These results demonstrated that the wettability of powders was enhanced with the addition of GMP. Overall, results derived from the wettability analysis indicated that the power samples prepared with the addition of GMP prior to drying had enhanced capability to interact with water compared to powder prepared without GMP addition or the original MCC-SD powder.

### 3.4. Kinetics of Powder Dispersion

**Figure 3:**
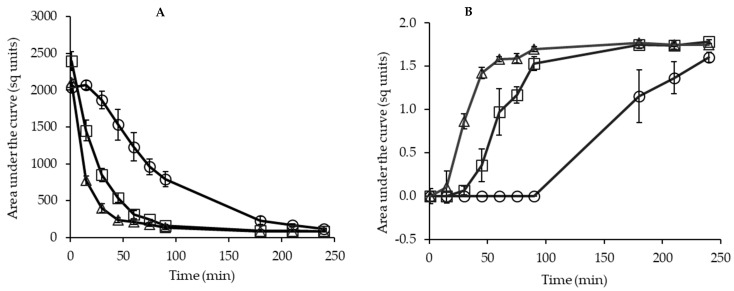
Distribution of area under the curve integrated from the peaks of volume density above 1 µm (**A**) and below 1 µm (**B**) during rehydration of powders: ○ P-MCC-0G, □ P-MCC-10G, and Δ P-MCC-20G mixed with 0, 10, and 20% GMP as a percentage of total protein, respectively. The error bars show the standard error.

The particle size distribution (PSD) of the powder samples was measured over 240 min of dynamic rehydration analysis. The particle size measured at 1 min after rehydration closely represents the average particle size of powders prior to rehydration. The volume-weighted mean particle size (D_4,3_) of powders P-MCC-0G, P-MCC-10G, and P-MCC-20G measured at 1 min of rehydration was in the range 132–156 μm, which is close to the size (120 μm) of MCC-SD original powder (Figure S3). The data for the PSD of GMP-SD powder could not be generated in this study as powders disintegrated rapidly in water. The value of D_4,3_ remained relatively constant for MCC-SD, whereas, for P-MCC-0G, P-MCC-10G, and P-MCC-20G, D_4,3_ decreased with increasing rehydration time up to 240 min (Figure S3). The decrease in particle size was due to disintegration of larger powder particles, with concomitant release of smaller particles [4], which is evident from the initial decrease in the size/volume of the peak of size range 1–1000 μm and the appearance of a peak in the size range 0.01–1 μm over time (Figure S4). To assess the kinetics of primary particle dispersion, we integrated the area under the curve of volume density as a function of particle size for primary powder particles and casein micelles separately and plotted as a function of rehydration time (Figure 3). The integrated area of primary powder particles decreased rapidly for P-MCC-10G and P-MCC-20G compared to P-MCC-0G, suggesting that the volume density of primary powder particles decreased due to particle disintegration as the rehydration progressed. Increasing the proportion of GMP in the powders resulted in a higher rate of particle disintegration (Figure 3A), with a concomitant increase in the area of casein micelles peak observed during rehydration (Figure 3B). The trend of increasing area across all powders progressed from an initial constant phase, through a rapid increase phase, and on to a plateau phase. P-MCC-20G had the shortest initial constant phase and a steeper/faster increase phase compared to P-MCC-10G, suggesting that a greater proportion of GMP in the former facilitated faster and more extensive disruption of larger powder particles into casein micelles. Although P-MCC-0G showed a decrease in particle size up to 90 min, the appearance of a peak related to casein micelles was not observed using this analytical method. This could be due to the volume density of primary powder particles being significantly higher for targeted obscuration during measurement, in which the volume fraction of casein micelles could be negligible. It is apparent that by the time the casein micelles area of P-MCC-20G reached the plateau phase, P-MCC-0G was at the initial constant phase. This suggests that there was a significant improvement in the dispersibility of powder particles prepared with GMP addition. While the focus of the study was not on comparing the data between original MCC-SD and freeze-dried powders, these results provided further evidence that freeze-dried powder rehydrates more quickly and that incorporation of GMP in MCC solution further enhances particle disintegration compared to original MCC-SD powder.

Various techniques have been previously explored for improving the dispersion characteristics of casein-rich powders. For example, dispersant with higher ionic strength (KCl, 80 mM), temperature (25–50 °C) and ultrafiltration milk permeate [28], calcium-binding agents (e.g., trisodium citrate or sodium hexametaphosphate) [14], or pH-mediated changes (up to pH 8.4) [30] have been shown to improve the dissolution of poorly soluble spray-dried MPC and milk protein isolate powders. On the other hand, emerging technologies such as hydrodynamic cavitation [31], ultrasonication [32], the use of CO_2_ to porosify MPC concentrate prior to drying [33], and high pressure (100 to 400 MPa) treatment of MPC solutions [34] have been explored for enhancing rehydration. However, such mechanical processes are energy-intensive and require an adjustment in process lines, adding capital expenditure and processing complexity [3]. Although alteration of chemical interactions between caseins by chelating calcium using sodium hexametaphosphate and maintaining casein integrity by treatment with polygalacturonase enzyme [15], pH adjustment to an alkaline range [29], or by dry blending MPI powder with sodium caseinate [35] show promising results for enhanced rehydration, these techniques involve chemical addition or generate non-micellar caseins that are normally undesirable for many applications. The results from the present study show the potential that formulation of MCC solutions with GMP enhances powder particle disintegration during rehydration through the use of dairy-based food ingredients with potential benefits from a nutritional perspective.

### 3.5. Casein and Glycomacropeptide Solubilization in Aqueous Phase during Powder Dispersion

**Figure 4:**
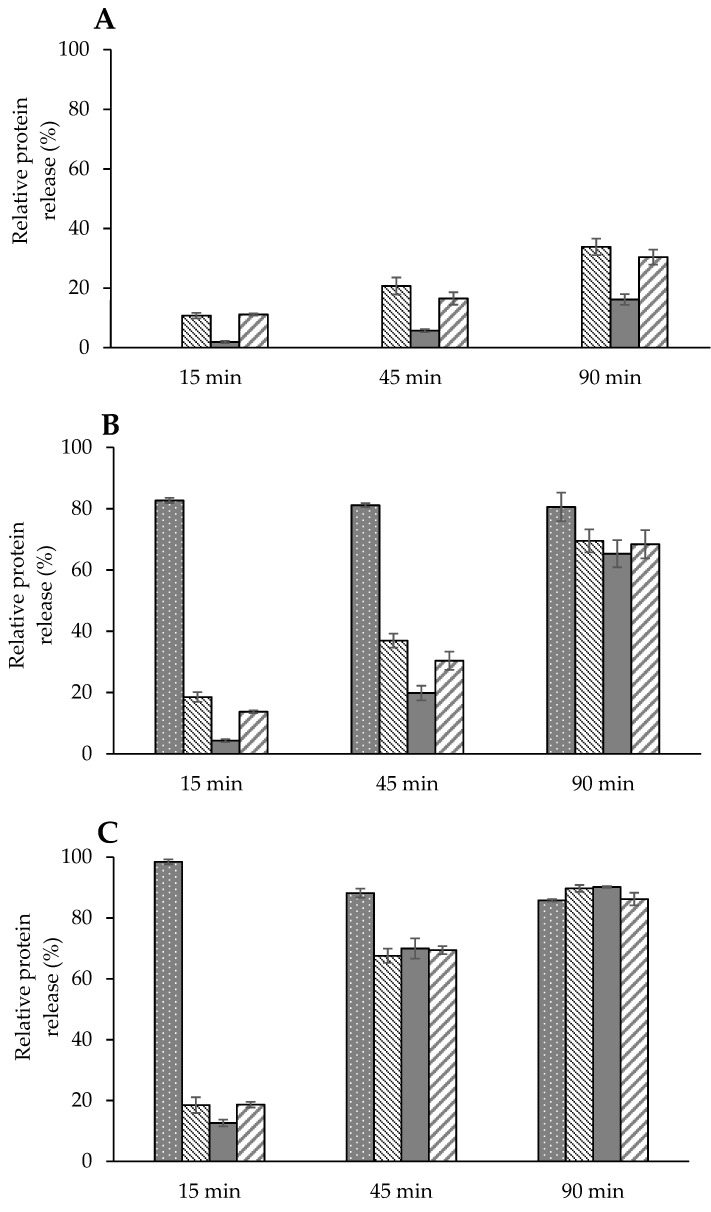
Relative release of proteins GMP (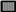), κ (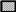), α (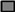), and β (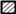) caseins in solution at 25 °C as a function of rehydration time. (**A**) Dispersion of powder containing no GMP (P-MCC-0G), (**B**) dispersion of powder containing 10% GMP (P-MCC-10G), or (**C**) 20% GMP (P-MCC-20G) as a percentage of total protein.

The relative release of proteins from powder particles into the dispersant solution at 15, 45, and 90 min, based on analysis of protein concentration in samples withdrawn after 24 h, is shown in Figure 4. In P-MCC-0G, protein solubility in terms of κ-, α-, and β-casein ranged between 11 and 34%, 2 and 16%, and 11 and 30%, respectively, during rehydration over 90 min. Both P-MCC-10G and P-MCC-20G, at 15, 45, and 90 min, had protein solubility values greater than P-MCC-0G. For P-MCC-10G, 83 to 81% GMP, 18 to 69% κ-casein, 4 to 65% α-casein, and 14 to 68% β-casein were detected in the same rehydration time. Similarly, for P-MCC-20G, 98 to 86% of GMP, 29 to 90% κ-casein, 13 to 90% α-casein, and 19 to 86% β-casein were identified. While caseins were diffusing slowly and progressively during rehydration, at 15 min of rehydration, both blended samples had more than 80% of GMP in the soluble form. The diffusion of GMP from powder particles into dispersant in the present study was independent of casein solubility, a trend which is similar to that observed with whey proteins diffusivity as analyzed in spray-dried MPC powders [10,36,37]. Faster dispersibility of P-MCC-10G or P-MCC-20G, compared to that of P-MCC-0G, supports the fact that development of insolubility in casein-dominant powder is principally due to the interactions of caseins within powder particles.

The extensive solubilization of GMP within the first 15 min of rehydration further supports the fact that the mobility of GMP within casein-dominant powder particles was not restricted, displaying characteristics similar to fast diffusing components, e.g., whey proteins and minerals [36]. Farias et al. [19] reported hydrodynamic diameter of GMP in the range 1 to 5 nm, with an average size being 2.5 nm at pH 6.7. Increase in aggregate size due to self-association of GMP molecules in MCC solution is less likely to occur towards neutral pH as GMP molecules acquire negative charge from Asp, Glu, and sialic acid, with net electrostatic repulsion, while the probability of hydrophobic interaction at domains AA1–5, 17–22, 35–39, and 58–65 would also be minimal [19]. Therefore, GMP in MCC solution is likely to be retained at the interface between caseins in their monomeric state, both in solution and powder form, exhibiting electrostatic repulsion between casein micelles. The lower negative values of zeta potential in the MCC solution blended with GMP (Table 1) further supports the fact that interparticle repulsion was greater in MCC-GMP powder blends. The rapid disintegration of P-MCC-10G and P-MCC-20G powders into caseins supports the hypothesis that GMP played the role in restricting interaction of caseins, as the ingress of water into powder particles was not determined to be a major factor contributing to powder disintegration, as shown by particles larger than water molecules being released shortly after rehydration [10,37].

## 4. Conclusions

This study investigated the influence of GMP on physicochemical properties of proteins in mixed MCC and GMP solutions and the rehydration characteristics of subsequent freeze-dried powders. Partial replacement of MCC with 10 or 20% GMP resulted in blended protein solutions with lower zeta potential, without influencing casein micelle size. The subsequent powders had enhanced wettability when compared to powders without inclusion of GMP. Blending of MCC with GMP to 20% of total protein resulted in rapid and extensive disintegration of primary powder particles, with faster and more extensive release of casein into solution. The current study demonstrated that the interactions between casein micelles can be modulated by GMP for improved rehydration characteristics of high-protein dairy powders, such as MCC. The results of this study provide novel insights in developing next-generation high-protein dairy powders with nutri-functional benefits associated with the added GMP.

## Figures and Tables

**Table 1:** Composition and physicochemical properties of MCC solutions mixed with 0 (S-MCC-0G), 10 (S-MCC-10G), and 20 (S-MCC-20G) % GMP as a percentage of total protein.

Parameter	S-MCC-0G	S-MCC-10G	S-MCC-20G
Total solids (%, *w*/*w*)	9.77 ± 0.11 ^a^	9.90 ± 0.13 ^a^	9.92 ± 0.14 ^a^
Protein (%, *w*/*w*)	8.64 ± 0.08 ^a^	8.58 ± 0.09 ^a^	8.59 ± 0.18 ^a^
Protein (%, *w*/*w*, dry basis)	88.5 ± 0.72 ^a^	86.7 ± 0.31 ^a^	86.6 ± 1.21 ^a^
Zeta potential (mV)	−25.8 ± 1.37 ^b^	−29.6 ± 1.97 ^ab^	−31.6 ± 1.09 ^a^

The values are mean ± standard deviation (*n* = 3). ^ab^ superscript letters, within a row, indicate statistically significant differences at a 95% confidence level.

## Supplementary Materials

The following are available online at https://www.mdpi.com/article/10.3390/foods10081960/s1, Figure S1: Distribution of casein micelle size in mixed micellar casein concentrate (MCC) and glycomacropeptide (GMP) protein solutions: ○ S-MCC-0G, □ S-MCC-10G, and Δ S-MCC-20G mixed with 0, 10, and 20% GMP as a percentage of total protein, respectively. Figure S2: Representative images of water droplet on the surface of powder tablets: MCC-SD (spray-dried original powder), P-MCC-0G (freeze-dried MCC powder without GMP addition), P-MCC-10G (freeze-dried MCC prepared with 10% GMP as a percentage of total protein), P-MCC-20G (freeze-dried MCC prepared with 20% GMP as a percentage of total protein), GMP-SD (original spray-dried GMP powder) at 50 S. Figure S3: Volume-weighted mean particle size (D_4,3_) of micellar casein powders: ○ P-MCC-0G, □ P-MCC-10G, and Δ P-MCC-20G obtained from micellar casein concentrate mixed with GMP prior to drying including original spray-dried MCC powder (● MCC-SD). The mean particle size values are shown as a function of rehydration time from 1 to 240 min and error bars represent standard error. Figure S4. Particle size distribution data of micellar casein powders: ○ P-MCC-0G, □ P-MCC-10G, and Δ P-MCC-20G obtained from micellar casein mixed with GMP at 0, 10, and 20% of total protein prior to drying. The PSD data are shown as a function of rehydration time: A (1 min), B (15 min), C (30 min), D (45 min), E (60 min), F (90 min), G (180 min), H (210 min), and I (240 min).
