# Supplementary material for: Influence of Glycomacropeptide on Rehydration Characteristics of Micellar Casein Concentrate Powder
Source: Foods. 2021 Aug 23;10(8):1960. doi: 10.3390/foods10081960 (PMC8394547; doi:10.3390/foods10081960)
Supplement: Supplementary file 1 [file foods-10-01960-s001.zip › foods-1352435-supplementary.pdf]

Supplementary data

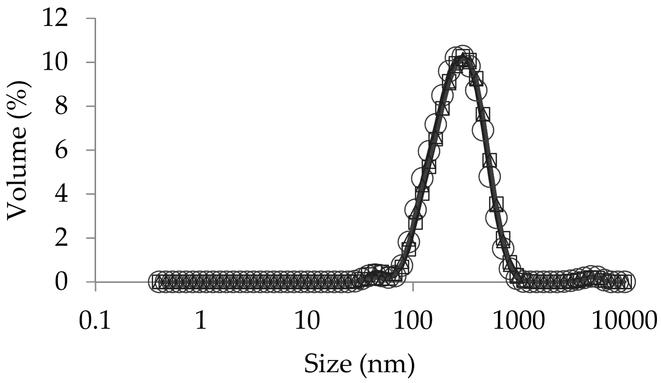

Figure S1. Distribution of casein micelle size in mixed micellar casein concentrate (MCC) and glycomacropeptide (GMP) protein solutions: ○; S-MCC-0G, □; S-MCC-10G, and Δ; S-MCC-20G mixed with 0, 10 and 20% GMP as % of total protein, respectively.

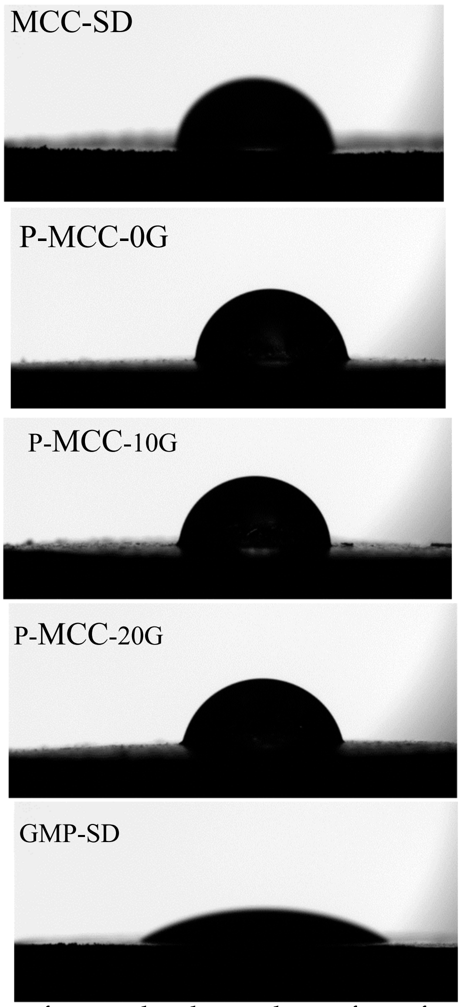

Figure S2. Representative images of water droplet on the surface of powder tablets: MCC-SD (spray-dried original powder), P-MCC-0G (freeze-dried MCC powder without GMP addition), P-MCC-10G (Freeze-dried MCC prepared with 10% GMP as a %of total protein), P-MCC-20G (freeze-dried MCC prepared with 20% GMP as a % of total protein), GMP-SD (original spray-dried GMP powder) at 50 S.

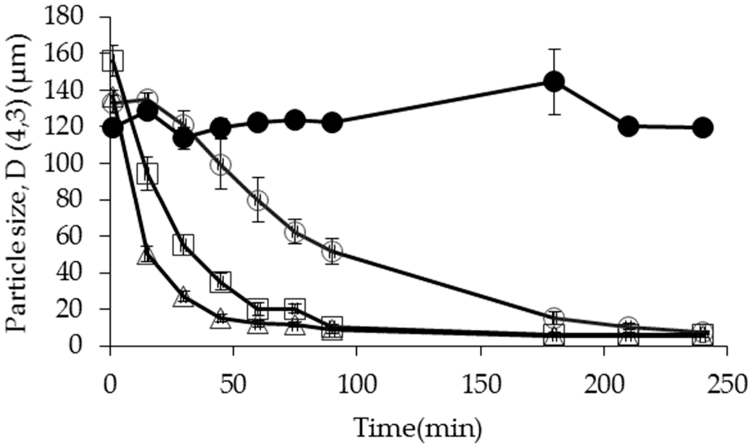

Figure S3. Volume-weighted mean particle size (D4,3) of micellar casein powders: ○; P-MCC-0G, □; P-MCC-10G, and Δ; P-MCC-20G obtained from micellar casein concentrate mixed with GMP prior to drying including original spray-dried MCC powder (●; MCC-SD). The mean particle size values are shown as a function of rehydration time from 1 to 240 min and error bars are standard error.

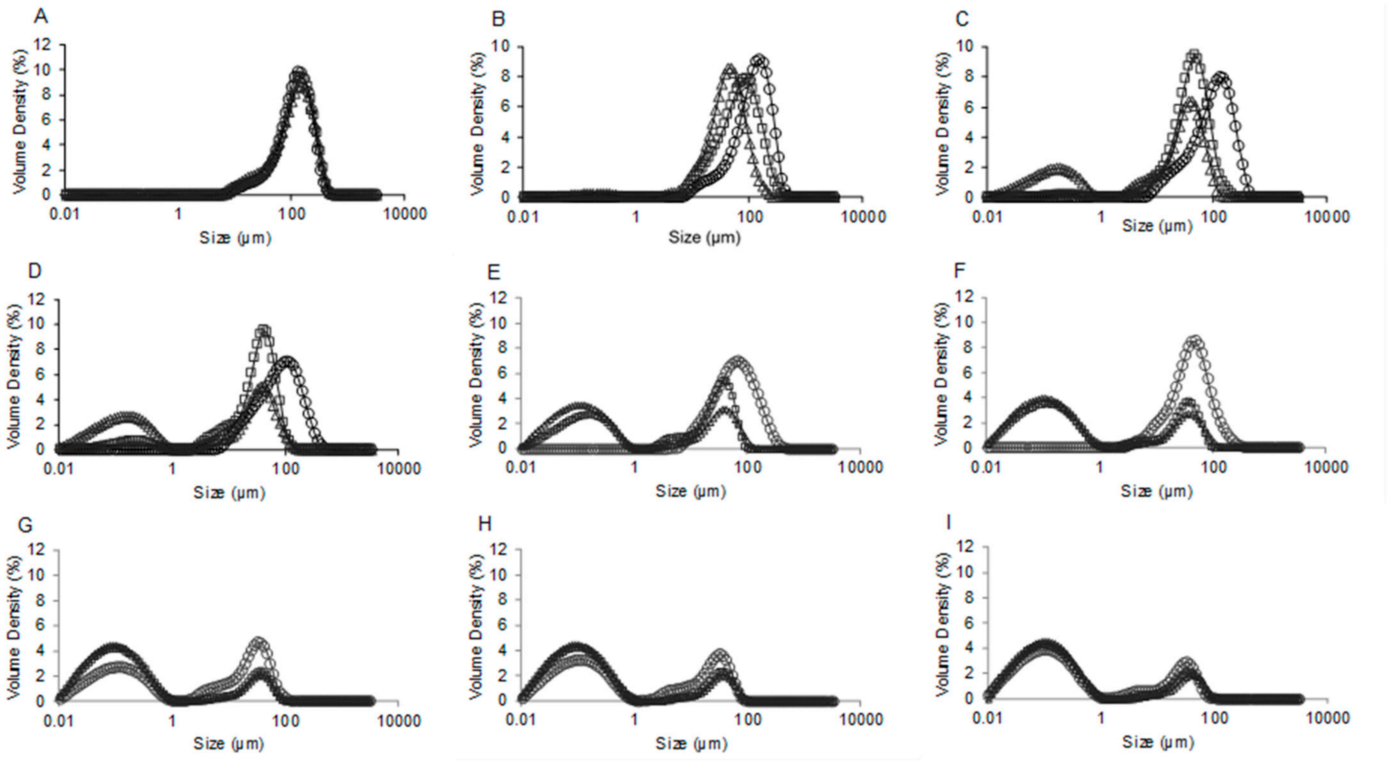

Figure S4. Particle size distribution data of micellar casein powders: ○; P-MCC-0G, □; P-MCC-10G, Δ; P-MCC-20G obtained from micellar casein mixed with GMP at 0, 10 and 20% as of total protein prior to drying. The PSD data are shown as a function of rehydration time: A (1 min), B (15 min), C (30 min), D (45 min), E (60 min), F (90 min), G (180 min), H (210 min) and I (240 min).
